# Gliopathy of Demyelinating and Non-Demyelinating Strains of Mouse Hepatitis Virus

**DOI:** 10.3389/fncel.2015.00488

**Published:** 2015-12-22

**Authors:** Lawrence C. Kenyon, Kaushiki Biswas, Kenneth S. Shindler, Manasi Nabar, Marjorie Stout, Susan T. Hingley, Judith B. Grinspan, Jayasri Das Sarma

**Affiliations:** ^1^Department of Pathology, Anatomy and Cell Biology, Thomas Jefferson UniversityPhiladelphia, PA, USA; ^2^Department of Biological Sciences, Indian Institute of Science Education and ResearchKolkata, India; ^3^Scheie Eye Institute and FM Kirby Center for Molecular Ophthalmology, University of PennsylvaniaPhiladelphia, PA, USA; ^4^Department of Neurology, Thomas Jefferson UniversityPhiladelphia, PA, USA; ^5^Department of Microbiology, Philadelphia College of Osteopathic MedicinePhiladelphia, PA, USA; ^6^Department of Neurology, Children's Hospital of PhiladelphiaPhiladelphia, PA, USA

**Keywords:** oligodendrocytes, astrocytes, mouse hepatitis virus, virus host attachment spike protein, demyelination, multiple sclerosis

## Abstract

Demyelination in the central nervous system induced by neurovirulent strains of Mouse Hepatitis Virus (MHV) is mediated by the viral spike glycoprotein, but it is not clear whether the mechanism of this disease pathology involves direct viral infection of oligodendrocytes. Detailed studies of glial cell tropism of MHV are presented, demonstrating that direct MHV infection of oligodendrocytes differs between demyelinating (RSA59) and non-demyelinating (RSMHV2) viral strains both *in vitro* and *in vivo*. Our results indicate that direct injury of mature oligodendrocytes is an important mechanism of virus-induced demyelination. *In vivo*, RSA59 infection was identified in spinal cord gray and white matter, but infected oligodendrocytes were restricted to white matter. In contrast, RSMHV2 infection was restricted to gray matter neurons and was not localized to oligodendrocytes. *In vitro*, RSA59 can infect both oligodendrocyte precursors and differentiated oligodendrocytes, whereas RSMHV2 can infect oligodendrocyte precursors but not differentiated oligodendrocytes. Viral spreading through axonal means to white matter and release of the demyelinating strain MHV at the nerve end is critical for oligodendrocytes infection and subsequent demyelination. Understanding the mechanisms by which known viruses effect demyelination in this animal model has important therapeutic implications in the treatment of human demyelinating disease.

## Introduction

Demyelination is the process by which axons lose their normal insulating myelin. Multiple sclerosis (MS) is a chronic, progressive, or relapsing and remitting demyelinating disorder that affects the central nervous system (CNS) specifically. In addition to demyelination, there is partial loss of oligodendroglial cells and axonal degeneration predominately within white matter, although some demyelination in MS also involves gray matter (Sharma et al., 2001; Bo et al., 2003; Vercellino et al., 2005; Lassmann et al., 2007; Rudick and Trapp, 2009). The mechanism of demyelination in MS is unknown, but may involve a T cell response triggered by virus infection (Allen and Brankin, 1993; Hernán et al., 2001; Hemmer et al., 2002). Despite numerous attempts to identify an etiologic agent, a specific virus has not been recognized. Various animal models have been developed to study MS, including experimental autoimmune encephalomyelitis (Kornek et al., 2000) as well as viral-induced CNS demyelinating disease models. One particularly useful mouse model utilizes MHV-induced demyelination that mimics the pathology of MS (Das Sarma, 2010).

Previous studies in our laboratory have analyzed the pathogenesis of recombinant MHV strains, RSA59 (demyelinating strain; DM), and RSMHV2 (non-demyelinating strain; NDM) that are isogenic except for the spike gene that encodes the virus-host attachment glycoprotein (Das Sarma et al., 2000, 2008, 2009). Both strains are capable of causing hepatitis, encephalitis, and meningitis, however, the two strains differ in their ability to induce subsequent demyelination and axonal loss (Das Sarma et al., 2009). Seven days post-infection, RSA59 produces demyelination that is best observed in the spinal cord. In contrast, RSMHV2 does not produce demyelination and only rarely demonstrates axonopathic changes in spinal cord white matter (Das Sarma et al., 2009). The inability of RSMHV2 to induce demyelination is due in part to a lack of transport of viral antigen (and the subsequent inflammatory reaction) to the white matter. Furthermore, *in vivo* and *in vitro* experiments demonstrate deficits in the ability of RSMHV2 to spread between neurons when compared to inter-neuronal spread by RSA59 (Das Sarma et al., 2009). RSA59-induced demyelination occurs in the setting of both axonal degeneration as well as macrophage mediated myelin stripping along intact axons (Das Sarma et al., 2009). While spike glycoprotein mediates spread of viral antigen to white matter through axonal transport, specific mechanisms leading to subsequent demyelination are not known. Viral antigen in white matter axons may be sufficient to trigger an inflammatory response that damages myelin. Alternatively, viral antigen may need to spread directly into oligodendrocytes, or indirectly to oligodendrocytes via astrocytes, using the spike protein.

Previously, another demyelinating strain, MHV-JHM, has been shown to produce a rapidly fatal encephalomyelitis, although some temperature sensitive mutants can produce non-fatal demyelination due to selective destruction of oligodendrocytes (Dubois-Dalcq et al., 1982). RSA59 or its parental demyelinating strain MHV-A59 can infect oligodendrocytes *in vitro*, isolated from either rat or mouse, or in oligodendrocyte cell lines (Liu et al., 2006, 2011; Li et al., 2010). Nevertheless, the inability of the RSMHV2 strain to induce demyelination has not been resolved and may be due to an inability to selectively destroy/impair oligodendrocytes in the spinal cord white matter. Alternatively, the failure of RSMHV2 to effect demyelination may be related to impaired axonal transport as has already been observed (Das Sarma et al., 2009). In the current study, viral tropism of RSA59 and RSMHV2 in spinal cord oligodendrocytes, myelin, neurons, and astrocytes is compared in order to further clarify the mechanism(s) of viral-mediated demyelination.

## Materials and methods

### Ethics statement

Use of animals and all experimental procedures were reviewed and approved by the Institutional Animal Care and Use Committee at the Philadelphia College of Osteopathic Medicine and Indian Institute of Science Education and Research-Kolkata (IISER-K). Animal protocols adhered to the guidelines of the United States National Institutes of Health Office of Laboratory Animal Welfare Guide for the Care and Use of Laboratory Animals, 8th Edition as well as CPCSEA, India.

### Viruses

Recombinant isogenic demyelinating (DM) strain of MHV, RSA59, and non-demyelinating (NDM) strain RSMHV2 were described in our previous studies (Das Sarma et al., 2002, 2008). RSA59 and RSMHV2 are isogenic except for the spike gene, which encodes an envelope glycoprotein that mediates many biological properties of MHV including viral attachment to host cells, as well as virus-cell and cell-cell fusion (Gallagher and Buchmeier, 2001). These recombinant strains also express enhanced green fluorescence protein (EGFP) (Das Sarma et al., 2002).

### Inoculation of mice

Four-week-old, MHV-free, C57BL/6 (B6) mice (Jackson Laboratories, Bar Harbor, Maine) were inoculated intracranially with 50% LD_50_ dose of RSA59 strain (20,000 PFU) or RSMHV2 (100 PFU) as described previously (Das Sarma et al., 2002, 2008). Ten mice were infected in each group.

Briefly, viruses were diluted in phosphate-buffered saline (PBS) containing 0.75% bovine serum albumin. Mice were anesthetized with 100 mg/kg ketamine and 10 mg/kg xylazine prior to inoculation. For intracerebral (i.c.) injection, 25 ml of diluted virus was injected directly through the skull into the left cerebral hemisphere. Mock-infected controls were injected similarly with an uninfected L2 cell lysate at a similar dilution. Mice were monitored daily for potential signs of disease including weight loss, waddling gait, partial limp, or lack of appetite. As with our prior studies, all mice developed minimal or no signs of disease, and mice were therefore sacrificed at time points based on the known disease course from prior histologic studies (Das Sarma et al., 2002, 2008). At the time of sacrifice, mice were anesthesized with ketamine/xylazine, then perfused transcardially with PBS, followed by cold PBS containing 4% paraformaldehyde and finally with 10 ml of PBS containing 10% sucrose. Spinal cord and brain tissues were removed and placed at 4°C for 4 h in 10% sucrose, followed by 30% sucrose overnight. Tissues were embedded in OCT medium (Tissue Tek, Hatfield, PA), sectioned sagitally with a microtome to 6 μm thickness, and mounted on glass slides.

Five mice were sacrificed at day 6 post-infection for viral titer estimation as well as routine paraffin based histopathological analysis and the rest were used for frozen sections. Cervical, thoracic and lumbar regions of spinal cord were harvested from each of 4 RSA59-infected and 5 RSMHV2-infected mice 6 days after inoculation. Four quadrants (dorsal/posterior column, anterior column, and two anterior horns) from two separate sections of each spinal cord level were examined. All together, we examined 96 quadrants for RSA59 and 120 quadrants for RSMHV2.

### Immunofluorescence microscopy

Immunofluorescence was performed on cryosections harvested from cervical, thoracic and lumbar regions of spinal cord from each of 4 RSA59-infected and 5 RSMHV2-infected mice 6 days after inoculation as described). For Olig2 staining, a Tyramide signal amplification kit (Molecular Probes CAT# T-20935; Invitrogen; Eugene, Oregon) was used. Primary antibodies and their dilutions to detect specific CNS cell types are listed in Table 1. Control slides were incubated in parallel with pre-immune rabbit sera, and sections from mock-infected mice were incubated with secondary antibodies only. Tissue sections were sequentially washed with PBS plus Triton X-100 and with PBS, then mounted and visualized by the Leica TCS SPE Confocal system. We examined 4 quadrants (dorsal column, ventral column, and two ventral horns) from two separate sections of each spinal cord level. All together we examined 96 quadrants for RSA59 and 120 quadrants for RSMHV2.

**Table 1:** **List of antibodies and their dilutions used for immunofluorescence**.

**Target cell/structure**	**Primary antibody**	**Primary antibody dilution**	**Fluorescent conjugated secondary antibody**	**Secondary antibody dilution**
Oligodendrocytes	Olig2, Rabbit Polyclonal anti-Olig2 antibody (Millipore Corporation, Billerica, MA)	1:500	Biotinylated Goat anti-Rabbit IgG (Jackson Immunoresearch, West Grove, PA)	1:100
Astrocytes	Rabbit polyclonal Anti-Glial Fibrillary Acidic Protein antibody (DAKO, Carpinteria, CA)	1:100	CY3-Goat anti-Rabbit IgG (Jackson Immunoresearch, West Grove, PA)	1:100
Myelin sheath	PLP (Proteo lipid protein) Rat IgG (Gift from Judith B. Grinspan, Children's Hospital of Philadelphia, Philadelphia, PA)	1:1	Texas Red-Goat anti- Rat IgG (Jackson Immunoresearch, West Grove, PA)	1:100
Axonal microtubule	Mouse monoclonal anti-Tau antibody (Sigma,)	1:100	TRITC-goat-anti-mouse IgG (Jackson Immunoresearch)	1:100
Dendritic microtubule	Mouse monoclonal anti-MAP2(2a + 2b) antibody (Sigma,)	1:100	TRITC-goat-anti-mouse IgG (Jackson Immunoresearch,)	1:100
Neurofilament	Mouse monoclonal, Mouse monoclonal anti-NFM (Neurofilament) antibody (Sigma)	1:100	TRITC-goat-anti-mouse IgG (Jackson Immunoresearch)	1:100
Synaptophysin	Mouse monoclonal	1:50	TRITC-goat-anti-mouse IgG (Jackson Immunoresearch)	1:100
Oligodendrocyte precursor cells	Mouse monoclonal anti-A2B5 antibody (Ref 21)	Neat	Rhodamine goat anti-mouse IgM (Jackson Immunoresearch)	1:100
Matured and differentiated oligodendrocytes	Mouse monoclonal anti-GalC galactocerebroside) antibody(Ref 21)	1:2	Rhodamine goat anti-mouse IgM (Jackson Immunoresearch)	1:100

For Olig2 staining, sections were specially post-fixed with ice-cold 95% ETOH at −20°C for 20 min. These were subsequently washed with 1X ice-cold TBS (Tris Buffer Saline) at room temperature (RT) for 10 min. Post-fixed tissues were processed as previously described (Das Sarma et al., 2008) for washing, permeabilization, and blocking prior to the addition of the anti-Olig2 antibody. Biotinylated anti-Rabbit IgG was used as secondary antibody and sections were incubated for 30 min at RT. Further staining steps were followed as per the Tyramide signal amplification kit manual. Briefly, sections were washed with 1X blocking buffer (10 mg/ml Component D) for 15 min and labeled with Strep-HRP (provided with the kit; 1:200 dilutions in diluted component D) at RT for 30 min with three consecutive 1X TBS washes for 5 min each. Sections were then stained with Tyramide (1:200 dilution in amplification buffer) for 10 min at RT, washed three times with 1X TBS for 5 min each and then mounted in Vectashield (Vector laboratories Inc; Burlingame, CA) with DAPI.

To determine the colocalization of RSA59 viral antigen to axonal cytoskeletal structures, sections were incubated for 2 h with monoclonal primary antibodies anti-TAU, anti-MAP (2A + B), anti-Neurofilament (NFM) and anti-Synaptophysin (Syn) to label the axonal microtubules, dendritic microtubules, axonal Neurofilament and synaptic terminals, respectively. Primary antibody incubation was followed by incubation with TRITC conjugated secondary goat anti-Mouse IgG for 1 h. All incubations were performed at 37°C in humidified chambers. Sections were mounted in Vectashield with DAPI.

All images were obtained with an Olympus IX81 fluorescence microscope. Images were acquired using Image ProPlus software with a 40X objective and Hamamatsu monochrome cool charged couple device camera. Images were taken in green and red excitation filters for EGFP and TRITC, respectively for each region and then merged with the help of the software.

### Isolation of oligodendrocyte-precursor cells (OPCs) from neonatal mouse brain

Primary cultures of mixed glial culture were prepared from day 0 newborn mice (Barres et al., 1992; Grinspan and Franceschini, 1995; Marek et al., 2008). Briefly, following the removal of meninges, brain tissues were minced and incubated in a shaking water bath at 37°C for 30 min in Hanks Balanced Solution (HBBS, GIBCO Life Technologies; Eugene, Oregon) in the presence of 300 μg/ml DNaseI and 0.25% trypsin (Sigma; St. Louis, MO). Enzyme digested dissociated cells were triturated with 0.25% fetal bovine serum (FBS), followed by a wash and centrifugation (300 × g for 10 min). The pellet was resuspended in HBBS, passed through a 70 μm nylon mesh, followed by a second wash and centrifugation (300 × g for 10 min). Following dilution with initial plating medium (Dulbecco's essential medium containing 1% penicillin-streptomycin, 0.2 mM L-glutamine and 10% FBS), cells were plated and allowed to adhere for 1 day in a humidified CO_2_ incubator at 37°C. After 24 h, any non-adherent cells were removed with HBBS without Ca and Mg and cells were switched to a serum free growth medium {Neurobasal medium containing B27 and 10 ng/ml and bFGF (Gibco Life Technologies, Eugene, Oregon) 2 ng/ml PDGF, and 1 ng/ml NT-3(PeproTech Inc. Rocky Hill, New Jersey}. Cultures were maintained in this medium until confluent (approximately 1 week). Oligodendrocyte progenitor cells (OPCs) present in mixed glial culture (mainly astrocytes and OPCs) were subjected to dislodging by a wash down method taking advantage of the differential adhesion properties of OPCs and astrocytes. OPCs mainly grow on top of the astrocyte layer and are less adherent than astrocytes. Dislodged oligodendrocytes were plated onto Poly-D-Lysine coated coverslips and maintained in serum free growth medium until the culture reached 80% confluence. Adherent cells were trypsinized and plated onto glass coverslips for cellular characterization.

Oligodendrocytes and Type-II astrocytes separated as described above were plated to determine viability and ability to grow in culture. In culture, they both grew well and exhibited the expected morphology. Purity of the culture was established using double label immunofluorescence with anti-GFAP and anti- A2B5 antibodies (markers for OPCs) as well as counterstaining with DAPI to detect cross contamination of GFAP-positive cells in OPC cultures and A2B5-positive cells in Type –II astrocyte cultures. Cultures were also stained with anti-CD11b antibody (microglia/macrophage marker) to look for microglia contamination, and routinely no CD11b positive cells were observed (data not shown).

### Differentiation of oligodendrocytes

Confluent OPCs were maintained in Oligodendrocyte Differentiation Medium {DMEM: F12 (1:1), 10% FBS supplemented with Transferrin (10 ug/ml), Insulin (5 μg/ml) and Sodium Selenite (30 nM) for 7–10 days. Day 7 differentiated oligodendrocyte cultures were stained with anti-GalC (Galactocerebroside), a marker of differentiated oligodendrocytes. Negative CD11b staining confirmed differentiated cultures remained free of microglia.

### Infection of primary OPCs and differentiated oligodendrocytes (DOs) with RSA59 and RSMHV2

On day 7 after explantation, OPCs or differentiated oligodendrocyte cultures were infected at a multiplicity of infection (MOI) of 1 with RSA59 and RSMHV2 or mock infected with non-infected cell lysate. After allowing viral adsorption for 1 h, cells were washed and placed in fresh specified media without virus. At 24 h after infection, cultures were immunolabeled with either anti A2B5 or anti-GalC. Details concerning antibodies and dilutions are shown in Table 1.

### Quantification of infected oligodendrocytes *in vivo* and *in vitro* by RSA59 and RSMHV2

Quantification of viral strain infectivity *in vivo* was determined by examination of 10 random spinal cord sections in which one hundred olig2 positive cells were counted by a blinded investigator. The percentage of virally infected olig2 positive cells in RSA59 and RSMHV2 infection was calculated. A similar strategy was used to quantify strain specific infection of PLP positive oligodendrocytes. *In vitro* quantification of strain specific infection of OPCs was determined by counting one hundred OPCs in 10 separate random tissue culture dish microscopic fields (20X). Statistical comparisons between the percentage of oligodendrocytes (OPCs or DOs) infected by RSA59 vs. RSMHV2 were made using unpaired two tailed student *t*-tests, with *p* ≤ 0.05 considered statistically significant.

## Results

### RSA59 (DM) and RSMHV2 (NDM) differ in their selective tropism for oligodendrocytes in gray and white matter

In order to elucidate the mechanism(s) of selective tropism of the RSA59 (DM) and RSMHV2 (NDM) strains, it is necessary to identify the types and distribution of cells within the CNS capable of supporting viral infection and transport. Four-week-old, MHV-free, C57BL/6 (B6) mice (Jackson Laboratory) were inoculated intracranially with either RSA59 or RSMHV2. MHV replication reaches its peak at day 5, and inflammation reaches its peak at day 7 post-infection. At day 5 post-infection, viral antigen from the RSA59 strain is localized to the spinal cord gray matter with subsequent spread to the adjacent white matter. At day 7 following RSA59 infection, viral antigen is mainly localized to the white matter (Das Sarma et al., 2008). In contrast, while at day 5 post-infection, the RSMHV2 strain viral antigen is transported to the spinal cord from the brain, viral replication and spread is primarily confined within the gray matter even at day 7 post-infection with infrequent spread to the white matter. Based upon the viral antigen dissemination kinetics, imaging was performed on spinal cord cryosections from day 6 post-infection when viral spread is maximal while cellular damage is limited. Since the viral strains used express EGFP, virus-infected cells were viewed directly by fluorescence microscopy. Identification of oligodendrocytes was performed using anti-sera to Olig2 (transcription factor for oligodendrocytes), usually present in oligodendrocyte nuclei. Detection was performed utilizing a red fluorescent Cy3-conjugated secondary antibody.

**Figure 1:**
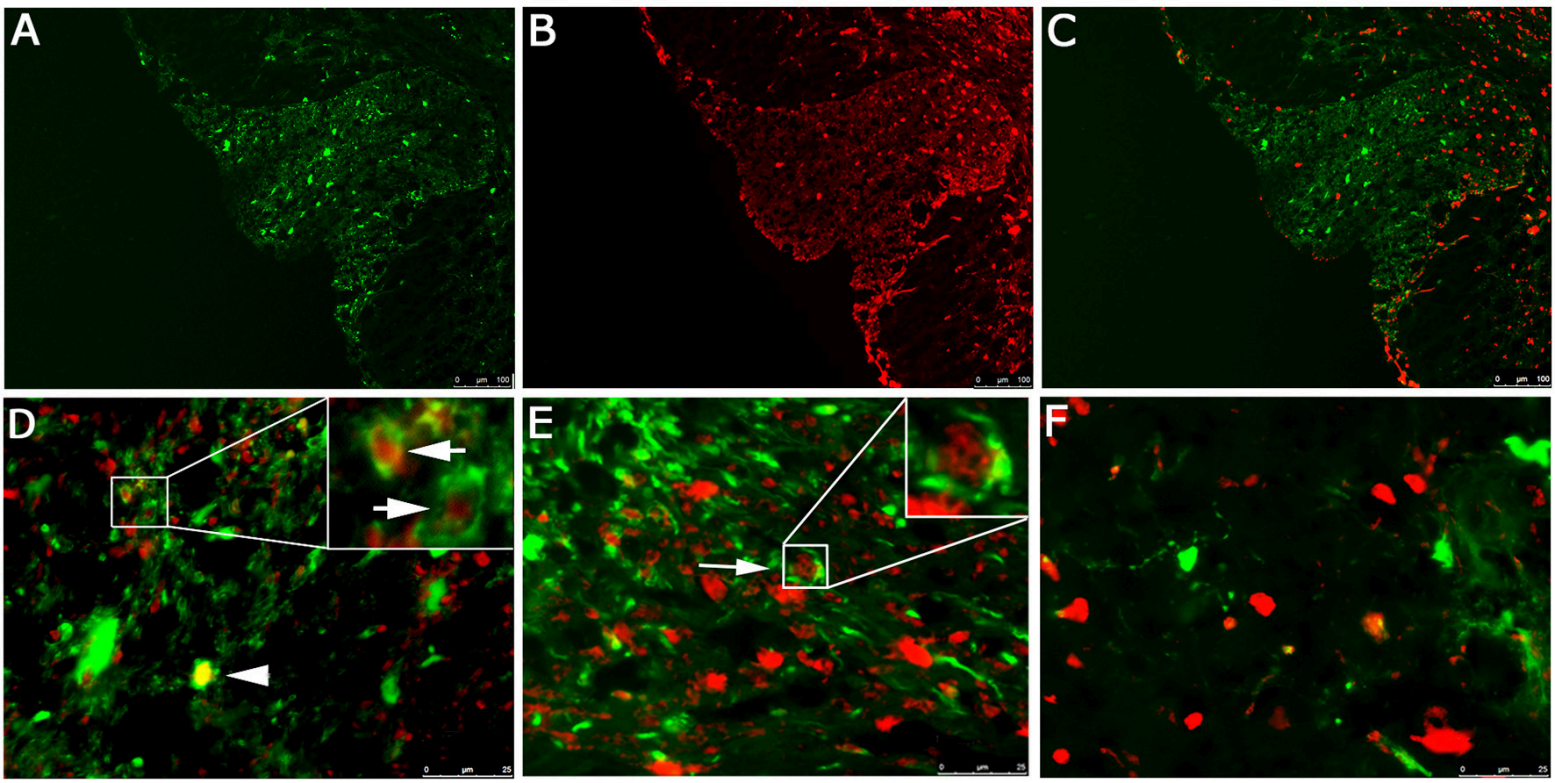
**Localization of RSA59 virus-infected cells and Olig2 in the spinal cord of infected mice at day 6 p.i**. Spinal cord dorsal columns were derived from mice infected with RSA59. A representative image showing EGFP fluorescence indicative of viral infection (green; **A**), and Olig2 labeling (red; **B**) does not demonstrate co-localization of EGFP and Olig2 in the merged image **(C)**. Dorsal columns white matter **(D)** and ventral horn white matter **(E)** reveal Olig2 positive nuclei surrounded by EGFP positive cytoplasm (arrows). Rare examples of co-localization are observed in (**D**, arrowhead). No co-localization or Olig2 positive nuclei surrounded by EGFP positive cytoplasm was observed in the gray matter **(F)**. (**A–C**, 100X; **D–F**, 630X).

EGFP fluorescence of spinal cord sections from RSA59 infected mice demonstrates infection of both gray (ventral horns) and white matter (dorsal and ventral columns). Olig2 staining observed in gray and white matter oligodendrocytes is primarily nuclear, whereas EGFP fluorescence is restricted to the cytoplasm (Figures 1A–C). Higher magnification images of the dorsal column white matter (Figure 1D, upper portion with inset) and gray/white matter junction (Figure 1E with inset) demonstrate red Olig2 nuclear fluorescence surrounded by EGFP cytoplasmic green fluorescence. These cells represent infected oligodendrocytes. Double fluorescence/immunofluorescence staining of the gray matter (Figure 1F) does not show Olig2 nuclear/EGFP cytoplasmic staining.

**Figure 2:**
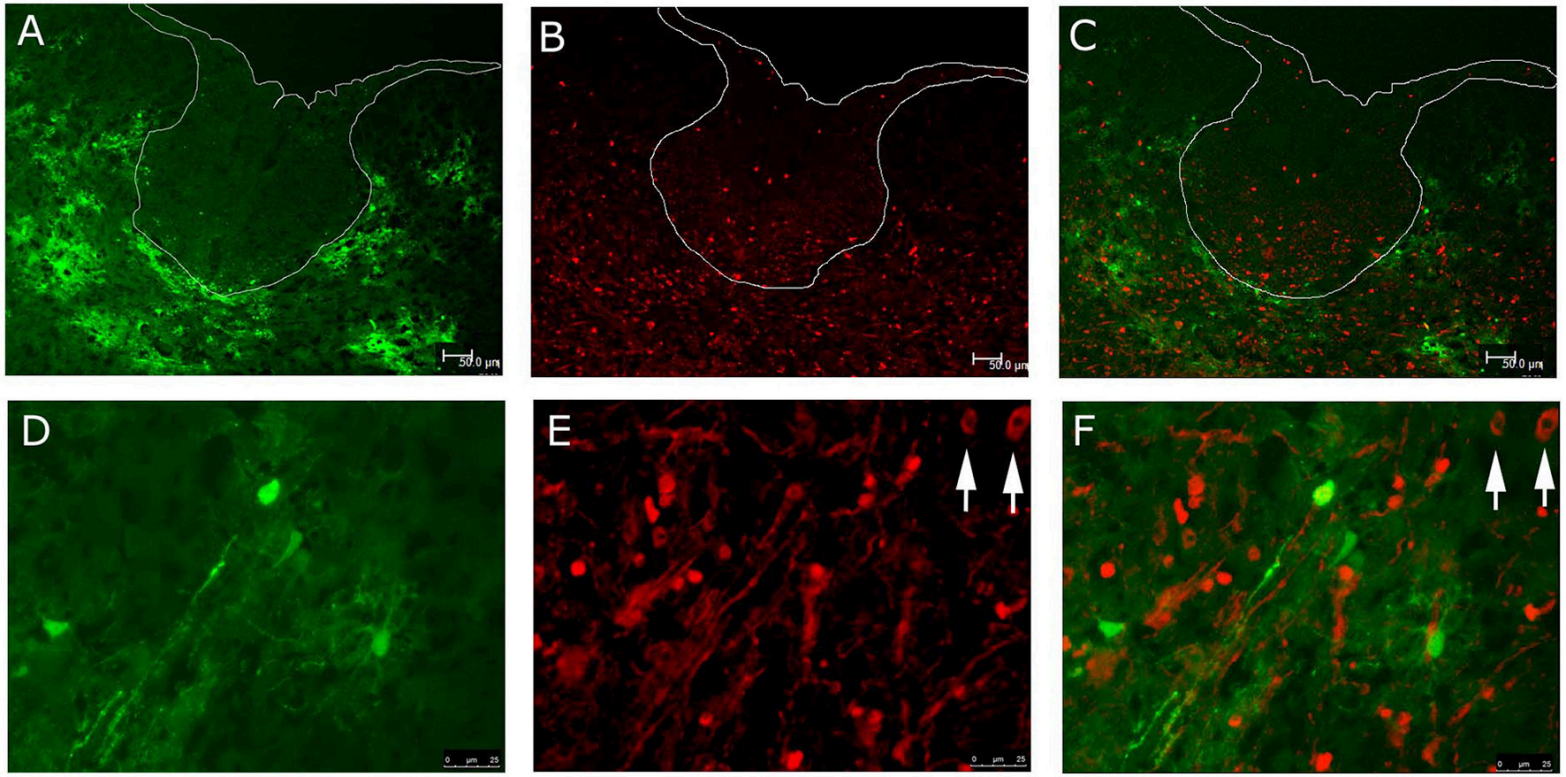
**Localization of RSMHV2 virus-infected cells and Olig2 in the spinal cord of mice at day 6 p.i**. EGFP fluorescence (green; **A**) indicating viral infection is restricted to spinal cord gray matter whereas Olig2 labeling (red; **B**) is present in both gray and white matter at day 6 p.i. Merged images are shown in **(C,F)**, while dorsal columns are outlined in white. Higher magnification images demonstrate a lack of co-localization of EGFP and Olig2 **(D–F)**. Arrows mark Olig2 positive nuclei in which there is an inhomogeneous distribution of Olig2. (**A–C**, 100X; **D–F**, 630X).

Similar staining was performed on spinal cord sections from RSMHV2 infected mice (Figure 2). Viral infection was restricted to the gray matter whereas Olig2 stained cells were observed in both gray and white matter. However, there were no examples of Olig2 nuclear/EGFP cytoplasmic fluorescence of cells in either gray or white matter as was observed in the white matter of RSA59 infected mice.

RSA59 strain is not only more efficient in producing widespread infection of gray and white matter; it also demonstrates increased localization to oligodendrocytes. Quantification of Olig2 immunofluorescence and EGFP revealed that 50% of Olig2 positive oligodendrocytes were infected by the RSA59 strain in the spinal cord white matter (see Quantification of Infected Oligodendrocytes *in vivo* and *in vitro* by RSA59 and RSMHV2 Section). For the purposes of this analysis, the dorsal columns and adjacent spinal cord gray matter were chosen since they were consistently identifiable at each level of the cord and showed the clearest and sharpest demarcation between gray and white matter.

### RSA59 and RSMHV2 differ in their ability to infect oligodendrocyte precursors (OPCs) and differentiated oligodendrocytes (DOs) *in vitro*

**Figure 3:**
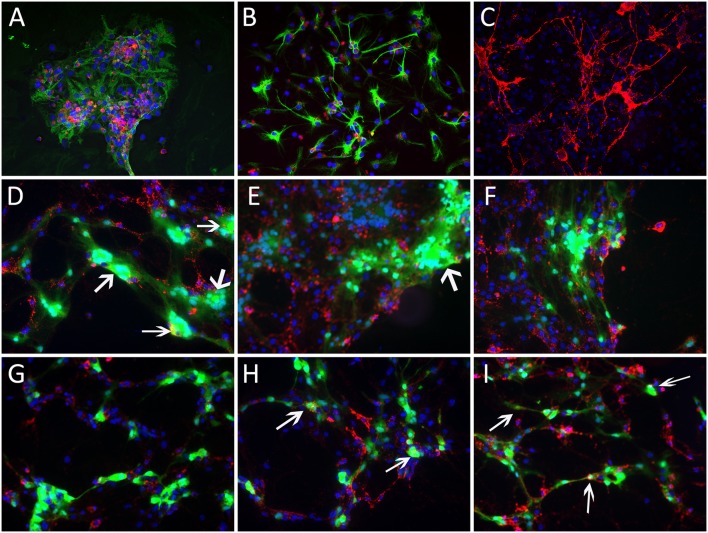
**Infection of oligodendrocyte precursors with RSA59 and RSMHV2**. Glial cells were isolated from neonatal C57Bl/6 mice and cultured in chemically defined medium specific for oligodendrocytes or oligodendrocyte precursors (OPCs). Seven day old mock infected mixed glial cells or cultures enriched for oligodendrocyte precursors or OPCs were double immunostained with anti A2B5 antibody, a marker for oligodendrocyte precursors (red) and anti GFAP, a Type-II astrocytic marker (green); nuclei were counterstained with DAPI (blue). Mixed glial culture **(A)** demonstrates labeling for both cell markers; cultures enriched for oligodendrocyte precursors or OPCs stain positive for TYPE-II astrocytes **(B)** or OPCs **(C)**, respectively. Three sets of mixed glial cultures enriched for OPCs were infected with RSA59 **(D–F)** or RSMHV2 **(G–I)** at a MOI of 1. Virus-infected cells were identified by EGFP (green). EGFP expression in A2B5 positive cells shows that RSA59 can infect OPCs and form syncytia that is characteristic of infection by the demyelinating MHV strain **(D–F)**. In RSMHV2-infected cultures, several cells were double positive for A2B5 (red) and EGFP (green), however, no syncytia were observed in RSMHV2-infected oligodendrocyte precursors **(G–I)**. Magnification (**A–I**: 600X). Arrows mark double positive cells.

**Figure 4:**
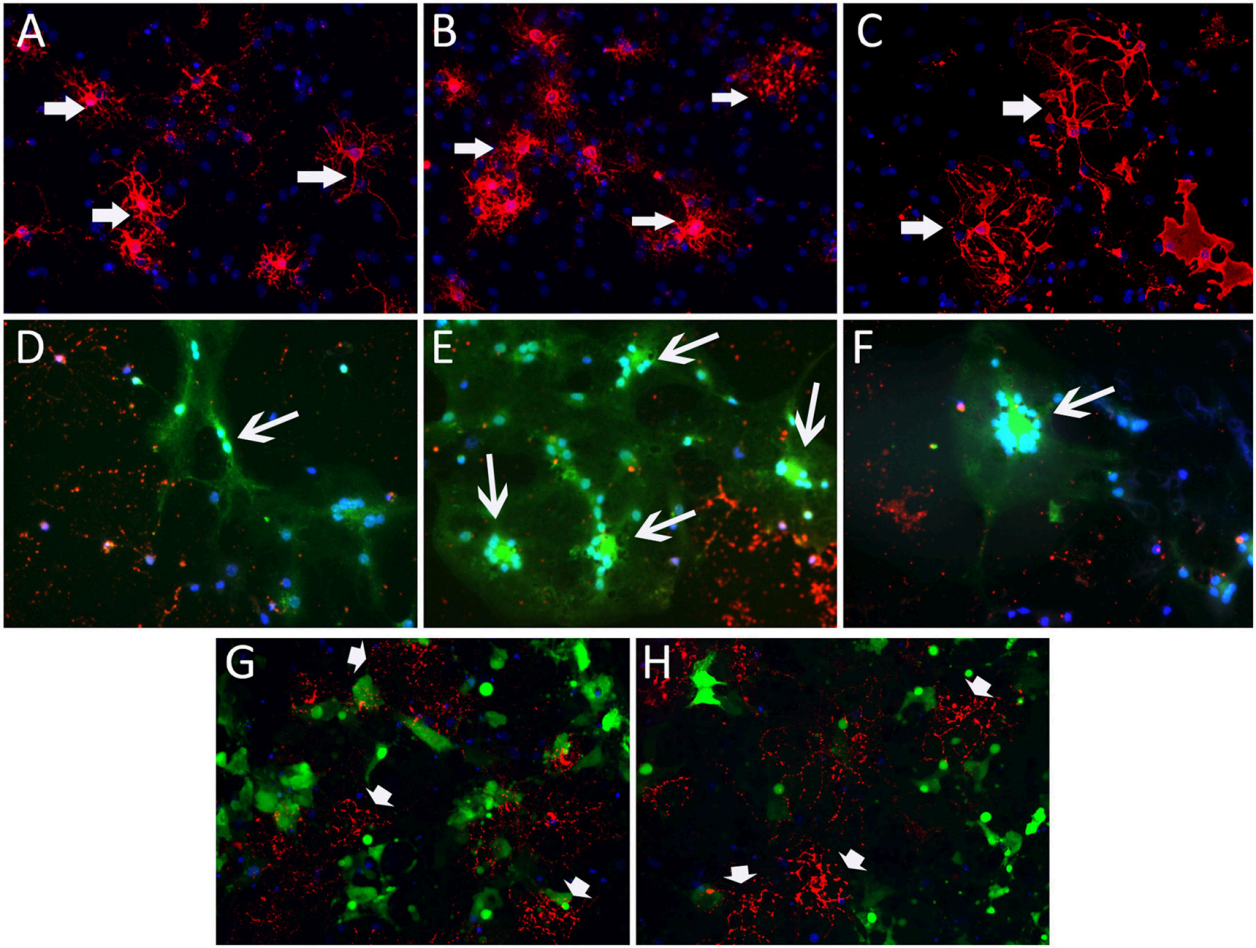
**RSA59 and RSMHV2 differ in their ability to infect differentiated mature oligodendrocytes**. Confluent oligodendrocyte precursor enriched cultures were maintained in oligodendrocyte differentiated medium for 7 days and infected with RSA59 or RSMHV2 at a M.O.I. of 1 for 24 h. Virus-infected cells were identified by EGFP (green). Differentiated oligodendrocytes were stained with anti-GalC antibody (marker for differentiated oligodendrocytes, red). Sister cultures of differentiated oligodendrocytes were either mock infected **(A–C)** or infected with RSA59 **(D–F)**, or with RSMHV2 **(G,H)**. In all three RSA59 infected cultures **(D–F)** syncytia formation is obvious and there are drastic reductions in the expression of GalC, but in RSMHV2 infected cultures, most of the infected cells were not oligodendrocytes. These data suggest that RSA59 can infect differentiated oligodendrocytes whereas RSMHV2 is unable to infect differentiated oligodendrocytes. Magnification (**A–H**: 600X). Thick arrows mark differentiated matured oligodendrocytes in **(A–C)**. Thin arrows in **(D–F)** mark infected mature oligodrndrocytes which formed syncitia. Arrowheads in **(G,H)** mark the differentiated oligodendrocytes which are not infected by RSMHV2.

We next examined the ability of the two MHV strains to infect isolated oligodendrocyte precursors as well as differentiated matured oligodendrocytes *in vitro*. Double label immunofluorescence with A2B5 (for OPCs) and GFAP (for astroctyes) or GalC (differentiated matured oligodendrocytes) confirmed that 60–70% of the cells either in oligodendrocyte precursor cultures or oligodendrocyte differentiated cultures were positive for A2B5 or GalC, respectively. Oligodendrocyte precursors were readily infected with both RSA59 and RSMHV2 whereas differentiated oligodendrocytes were only infected by RSA59. In both precursor cells and differentiated oligodendrocytes, RSA59 forms syncytia. In contrast, RSMHV2 infected cells were unable to form syncytia. In both cases, viral infection is associated with a dramatic reduction in GalC expression. This is the first demonstration that RSA59 and RSMHV2 strains have differential oligodendroglial tropism both *in vitro* and *in vivo* (Figures 3, 4).

### Both RSA59 and RSMHV2 are unable to infect astrocytes in the spinal cord

**Figure 5:**
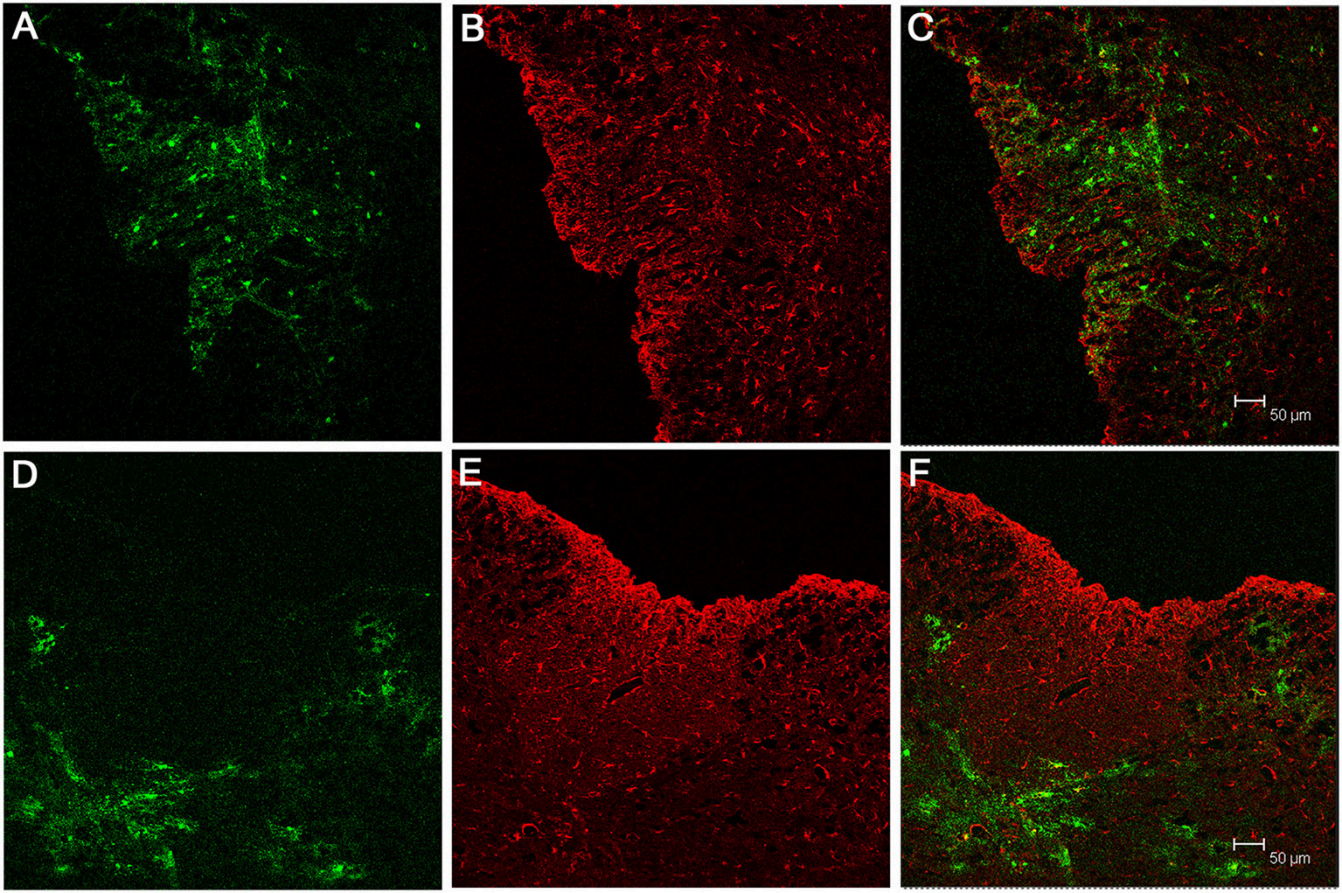
**EGFP expression in RSA59 or RSMHV2 infected cells does not co-localize with astrocytes in the spinal cord**. Mice were infected with either RSA59 **(A–C)** or RSMHV2 **(D–F)**. EGFP fluorescence (green; **A,D**) identifies virus infected cells whereas labeling with anti-GFAP (red; **B,E**) identifies astrocytes. Merged images **(C,F)** do not demonstrate EGFP expression in astrocytes in RSA59 or RSMHV2 infected mice. All images 100X.

Despite the facility by which the two MHV stains infect oligodendrocyte precursors *in vitro*, they are unable to infect spinal cord astrocytes *in vivo*. Immunofluorescence for GFAP, a specific marker of glial cells, especially astrocytes, was present throughout the spinal cord gray and white matter in RSA59 and RSMHV2 infected mice, however double fluorescence/immunofluorescence staining failed to demonstrate co-localization of virus and astrocytes (Figure 5).

### RSA59 but not RSMHV2 can spread to the myelin sheath

**Figure 6:**
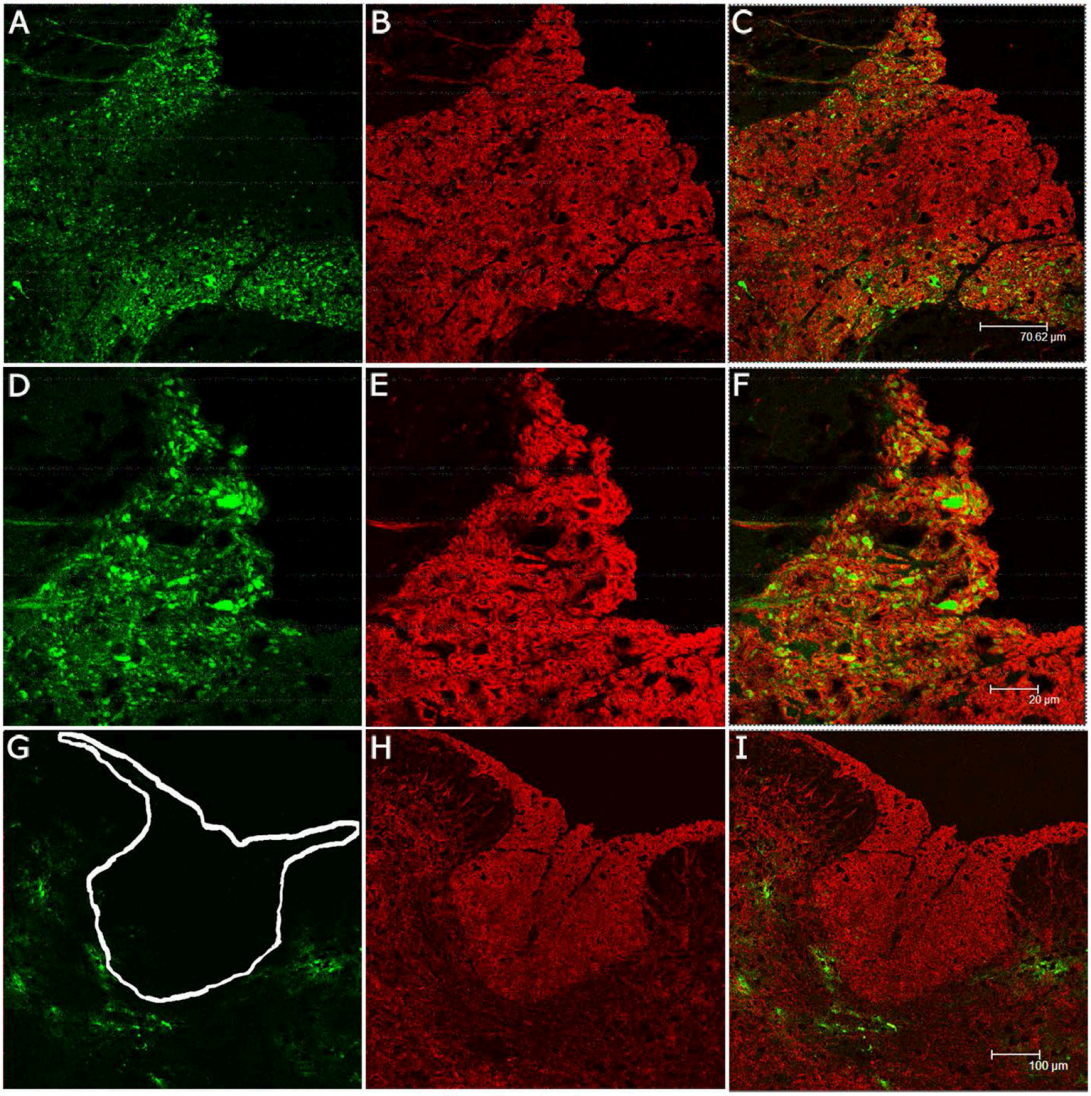
**Co-localization of EGFP- and myelin (PLP) in spinal cords of mice infected with RSA59 or RSMHV2**. Mice were infected with RSA59 (**A–C**, 100X; **D–F**, 200X) or RSMHV2 (**G–I**, 100X). EGFP fluorescence (green) identifies virus infected cells **(A,D,G)** while anti-PLP labeling (red) was used to detect PLP in the myelin sheath **(B,E,H)**. Merged images of spinal cord sections from RSA59-infected mice **(C,F)** demonstrate EGFP positive axons and co-localization of PLP and EGFP in the surrounding myelin sheaths. However, no co-localization of PLP and EGFP is observed in spinal cord sections from RSMHV2-infected mice **(I)**. The white outline in **(G)** marks the location of dorsal columns.

Infection of oligodendrocyte cell bodies does not necessarily assure viral antigen spread into the myelin sheath. Therefore, we performed double fluorescence/immunofluorescence staining of the dorsal columns utilizing EGFP expression by infected cells and proteolipid protein (PLP), a protein associated with CNS myelin (Figure 6). PLP fluorescence is observed in both RSA59 and RSMHV2 infected mice. In RSA59 infected mice, the EGFP signal is predominately in an axonal pattern with surrounding PLP fluorescence. However, there is some colocalization within the myelin sheath as well. No EGFP axonal staining or double labeling is detected after RSMHV2 infection. While some EGFP signal is observed in the gray matter, there is no double labeling following infection with either strain. Therefore, the RSA59, but not the RSMHV2 strain is capable of intra-axonal transport with spread to the myelin sheath.

### RSA59 viral antigen axonal spread to spinal cord white matter

**Figure 7:**
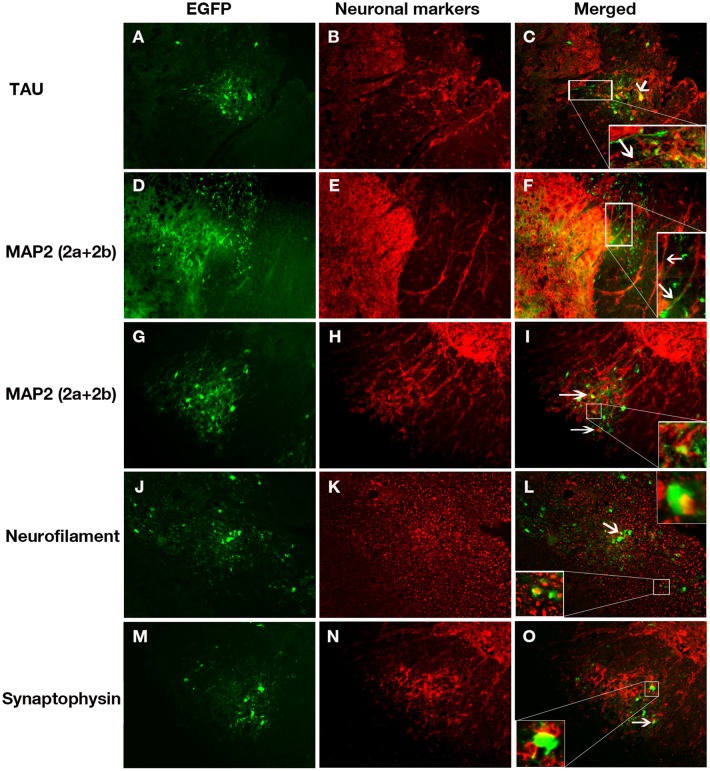
**Immunolabeling of RSA59 infected spinal cord sections with different neuronal specific markers**. Virus-infected cells (green; **A,D,G,J,M**) can be seen in posterior columns and adjacent dorsal gray matter **(A–F)** or ventral horn region **(G–O)** of spinal cords from mice infected with RSA59. Cells immunolabeled for Tau **(B)**, Map2 (2a + 2b) **(E,H)**, Neurofilament **(K)**, and synaptophysin **(N)** are shown in red. Merged images demonstrating viral infected cells and cells with different specific antibody labeling are shown in **(C,F,I,L,O)**. The insets show magnified views of the co-localization of RSA59 infected cells with the neuronal cytoskeletal markers. All magnifications are 630X.

In order to further confirm that RSA59 viral antigen spreads to spinal cord white matter via axons, serial double immunofluorescence was performed with axonal microtubule marker Tau; dendritic microtubule markers MAP2(2a + 2b), neurofilament, and synaptophysin (presynaptic vesicle membrane glycoprotein). As can be seen in Figure 7, viral antigen (EGFP) colocalizes with neurons and their axonal microtubules in the spinal cord white matter. Merged Tau immunofluorescence images with EGFP demonstrates colocalization in the white matter and the region of the gray-white matter junction (Figure 7C), demonstrating that viral antigen is present in the axons of infected neurons. Similarly, MAP2(2a + 2b) immunofluorescence, a marker of the somatodendritic compartments of neurons, colocalizes with EGFP (Figures 7F,I). Note that MAP2 is also expressed in some axons and EGFP positive axons crossing through the gray matter from the posterior columns demonstrate doubly labeled axons (Figure 7F). Neurofilament antibody labeled the neuronal cell bodies and axons in the spinal cord (Figure 7K). In the white matter of the spinal cord, there are several examples of Neurofilament immunofluorescence colocalizing with EGFP (Figure 7L). Anti-Synaptophysin antibody marks presynaptic vesicles. RSA59 viral antigen positive cells partially colocalized with synaptophysin (Figure 7O).

### Quantification of virally infected oligodendrocytes *in vivo* and *in vitro*

**Figure 8:**
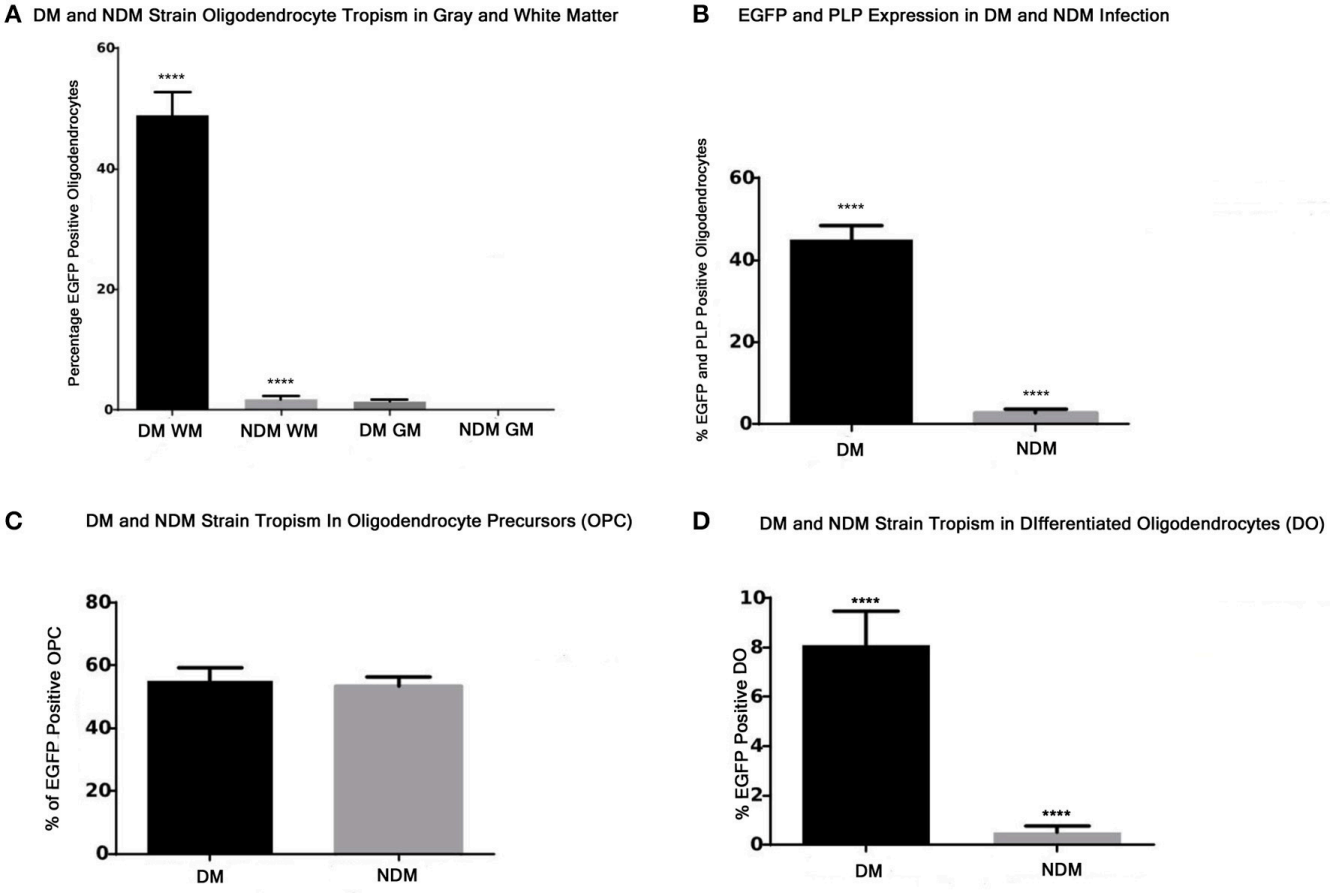
**Quantification of infected oligodendrocytes ***in vivo*** and ***in vitro*** by RSA59 and RSMHV2. (A)** Percentage of infected (EGFP positive) oligodendrocytes (olig2 positive) in DM and NDM infection 6 days p.i. Ten random spinal cord sections were examined from mice infected with RSA59 (DM) or RSMHV2 (NDM) strains. One hundred olig2 positive cells were counted and the percentage of olig2 positive cells also positive for viral antigen (EGFP) was determined in both gray (GM) and white matter (WM). **(B)** Percentage of infected (EGFP positive) oligodendrocytes (PLP positive) in DM and NDM infection 6 days p.i. Ten random spinal cord sections were examined. One hundred PLP positive cells were counted and the percentage of PLP positive cells also positive for viral antigen (EGFP) was determined. **(C)** Percentage of DM and NDM infected (EGFP positive) oligodendrocyte precursors (OPC) *in vitro*. Cultures were infected with DM or NDM strain at a MOI of 1 for 24 h. One hundred OPCs were counted in 10 separate random fields in tissue culture dishes and the percentage of EGFP positive OPCs was determined. **(D)** Percentage of DM and NDM infected (EGFP positive) differentiated oligodendrocytes (DO) *in vitro*. Cultures were infected with DM or NMD strain at a MOI of 1 for 24 h. One hundred DOs were counted in 10 separate random fields in tissue culture dishes and the percentage of EGFP positive DOs was determined. Statistical comparisons between the percentage of oligodendrocytes (OPCs or DOs) infected by RSA59 vs. RSMHV2 were made using unpaired two tailed student *t*-tests. Asterisks indicate significant differences (^****^p < 0.001).

The statistical significance of viral strain infectivity *in vivo* and *in vitro* was determined by examination of random spinal cord sections or tissue culture fields in which one hundred olig2 positive cells were counted by a blinded investigator as described in the Materials and Methods Section. Quantitative analysis of the relative infectivity of RSA59 (DM) and RSMHV2 (NDM) demonstrates that RSA59 strain infects 50% of olig2 positive oligodendrocytes in white matter (*P* < 0.005) and very few in gray matter. In contrast, RSMHV2 infects less than 2% of oligodendrocytes in white matter and none in gray matter (Figure 8A). Quantitative analysis of the relative infectivity of RSA59 (DM) and RSMHV2 (NDM) demonstrates that RSA59 strain infects 45% of PLP positive oligodendrocytes. In contrast, RSMHV2 infects less than 1% of oligodendrocytes (Figure 8B; *P* < 0.005).

Quantitative analysis of the infectivity of RSA59 (DM) and RSMHV2 (NDM) demonstrates that both RSA59 and RSMHV2 strains infect 50–60% of oligodendrocyte precursors (OPC) (Figure 8C). In contrast, there is a significant difference (more than 10-fold) in the ability of RSA59 and RSMHV2 to infect differentiated oligodendrocytes (DO; *P* < 0.005; Figure 8D).

## Discussion

Isogenic MHV strains RSA59 and RSMHV2 are both capable of causing hepatitis, encephalitis, and meningitis, but RSMHV2 is unable to effect subsequent demyelination (Das Sarma et al., 2008, 2009). Following viral infection, axonal damage can occur concurrently with and independently of demyelination and direct viral-mediated axonal damage can occur as a primary pathology, separate from demyelination. Despite the fact that both strains can infect neurons, there is impaired inter-neuronal spread of the RSMHV2 strain *in vivo* and *in vitro* as well as a lack of translocation from gray matter to white matter (Das Sarma et al., 2009) in the spinal cord. Strain specific viral dissemination and selective replication in CNS cells are also critical for MHV induced neuropathology. Our studies demonstrated that different doses of the viral strains (RSA59, 20,000 PFU vs. RSMHV2, 100 PFU) do not affect astrocyte infection within the brain (Das Sarma et al., 2008). The two very distinct and different doses of viruses used are based on 50% of the LD50, where previously it has been demonstrated that both strains replicate at a similar rate in the brain and spinal cord and viral titers at day 5 and day 7 post-infection are almost the same (Das Sarma et al., 2000, 2009). Moreover, even at 100 PFUs inoculation, RSA59 can cause demyelination although the number and size of demyelinating plaques formed was less compared to an inoculum of 20,000 PFUs (Das Sarma et al., 2000).

The current differential oligodendroglial tropism studies both *in vitro* and *in vivo* suggest that direct oligodendrocyte infection may be a requirement for MHV to promote demyelination. RSMHV2 infection was restricted to the gray matter whereas Olig2 stained cells were observed in both gray and white matter. No colocalization was observed even in the gray matter. Furthermore, there were no examples of Olig2 nuclear/EGFP cytoplasmic fluorescence of cells as was observed in the RSA59 infected mice. *In vitro*, oligodendrocyte precursors were infected by both strains whereas differentiated oligodendrocytes were only infected by the DM strain, suggesting that one needs to infect the mature oligodendrocytes, cells capable of producing myelin, in order to cause demyelination. Infection of OPCs might, however, prevent remyelination following demyelination as the OPCs are likely to be the cell type of choice for remyelination. Moreover, RSA59 strain not only infected oligodendrocytes, but also caused formation of syncytia. In contrast, RSMHV2 infected oligodendrocytes were unable to form syncytia. Taken together, our data support direct infection of oligodendrocytes as a potentially critical factor for viral induced demyelination.

In addition to virus being found in oligodendrocyte cell bodies, our results show by double labeling with EGFP and anti-PLP immunofluorescence that the RSA59 strain localizes to axons whereas the RSMHV2 strain does not. This lack of transport of the RSMHV2 strain to spinal cord white matter may explain its failure to induce demyelination. Immunofluorescence and colocalization studies with neuronal cytoskeleton specific markers also demonstrate that RSA59 strain colocalizes with axonal microtubules, dendritic microtubules and neurofilament protein as well as presynaptic vesicles (synaptophysin). The latter result provides evidence for a mechanism by which RSA59 can spread transynaptically to the spinal cord white matter and gain access to oligodendrocytes and the myelin sheath. In a recent study, we also demonstrated that RSA59 can directly infect and activate microglia in the spinal cord white matter (Chatterjee et al., 2013).

In the brain, viral infection of oligodendrocytes was identified following infection with both the RSA59 and RSMHV2 (unpublished data), whereas current data in the spinal cord shows oligodendrocyte infection only with RSA59. This differential viral tropism between the brain and spinal cord is likely due to the route by which virus gains access to the white matter. Mice are infected by transcranial inoculation, a traumatic disruption of the gray-white matter interface that permits viral particles direct access to myelin. From the myelin sheath, viral particles could then spread proximally to the oligodendrocyte cytoplasm. In contrast, viral involvement of the spinal cord occurs non-traumatically. A major mechanism of viral spread to spinal cord white matter is through spike-mediated axonal transport (Das Sarma et al., 2009), which may partly explain the inability of RSMHV2 viral antigen to enter oligodendrocytes in the spinal cord. Our results demonstrate that even though RSMHV2 virus reaches spinal cord gray matter neurons and replicates (Das Sarma et al., 2008), it does not spread to oligodendrocytes or myelin. Thus, spike glycoprotein must also play a significant role in facilitating direct spread of virus from neurons to oligodendrocytes.

The overall pattern of co-localization of viral antigen in specific CNS cell types provides a logical mechanism for MHV infection of the spinal cord. Brain neurons can be infected with both RSA59 and RSMHV2 following intracranial inoculation. Virions are likely unrestricted within neuronal cytoplasm and therefore able to diffuse or be actively transported through the axoplasm into spinal cord gray matter. From the axoplasm, only the RSA59 virus is then capable of spreading to spinal cord neurons and being transported anterogradely to the white matter, or of crossing into the myelin sheath and from there, into the oligodendrocyte cytoplasm. The lack of co-localization with GFAP with either strain indicates that the spread of MHV infection in the spinal cord does not involve an indirect route to oligodendrocytes through astrocytes. The lack of viral infection in spinal cord astrocytes is unexpected since our previous work (Das Sarma et al., 2008) clearly showed that both RSA59 and RSMHV2 could infect brain astrocytes *in vivo*. As described above, there was a similar discrepancy in the colocalization of EGFP in brain vs. spinal cord oligodendrocytes due to the different routes of infection (unpublished data). Traumatic transcranial inoculation likely provides viral access to astrocytes due to disruption of the pia and blood/brain barrier whereas no such disruption occurs in the spinal cord. Our results are similar to those seen with other viral strains; direct viral induced oligodendroglial dystrophy is a common etiology of JC virus induced demyelination (Weiner et al., 1972; Major et al., 1992).

Despite strong suspicions of a viral etiology, no specific virus has been identified that is responsible for MS in humans. Understanding the mechanisms by which known viruses effect demyelination in an animal model has important therapeutic implications in the treatment of human demyelinating disease. Our experimental system is a valuable tool in this regard and, more generally, in the elucidation of the mechanisms of how neurotropic viral infection can directly lead to oligodendrogliopathy in demyelination.

## Author contributions

MN, MS, and JD performed all animal experiments and immunohistochemistry. KB participated animal experiments in India and did neuronal staining of MHV infected tissues. LK and JD participated in data analysis and data interpretation and drafted the manuscript. LK blindly read the pathological samples. KS, SH, and JG were involved in critical revisions of the manuscript. JD led all aspects of this work including experimental design, participated in or supervised all experimental procedures, analyzed, and interpreted data and critically revised the manuscript.

### Conflict of interest statement

The authors declare that the research was conducted in the absence of any commercial or financial relationships that could be construed as a potential conflict of interest.

## References

[B1] Allen I. Brankin B. (1993). Pathogenesis of multiple sclerosis–the immune diathesis and the role of viruses. J. Neuropathol. Exp. Neurol. 52, 95–105. 10.1097/00005072-199303000-00001 8440999

[B2] Barres B. A. Hart I. K. Coles H. S. Burne J. F. Voyvodic J. T. Richardson W. D.. (1992). Cell death and control of cell survival in the oligodendrocyte lineage. Cell 70, 31–46. 10.1016/0092-8674(92)90531-G 1623522

[B3] Bo L. Vedeler C. A. Nyland H. I. Trapp B. D. Mørk S. J. (2003). Subpial demyelination in the cerebral cortex of multiple sclerosis patients. J. Neuropathol. Exp. Neurol. 62, 723–732. 12901699 10.1093/jnen/62.7.723

[B4] Chatterjee D. Biswas K. Nag S. Ramachandra S. G. Das Sarma J. (2013). Microglia play a major role in direct viral-induced demyelination. Clin. Dev. Immunol. 2013:510396. 10.1155/2013/510396 23864878 PMC3705805

[B5] Das Sarma J. (2010). A mechanism of virus-induced demyelination. Interdiscip. Perspect. Infect. Dis. 2010:109239. 10.1155/2010/109239 20652053 PMC2905936

[B6] Das Sarma J. Fu L. Tsai J. C. Weiss S. R. Lavi E. (2000). Demyelination determinants map to the spike glycoprotein gene of coronavirus mouse hepatitis virus. J. Virol. 74, 9206–9213. 10.1128/JVI.74.19.9206-9213.2000 10982367 PMC102119

[B7] Das Sarma J. Iacono K. Gard L. Marek R. Kenyon L. C. Koval M.. (2008). Demyelinating and nondemyelinating strains of mouse hepatitis virus differ in their neural cell tropism. J. Virol. 82, 5519–5526. 10.1128/JVI.01488-07 18385249 PMC2395180

[B8] Das Sarma J. Kenyon L. C. Hingley S. T. Shindler K. S. (2009). Mechanisms of primary axonal damage in a viral model of multiple sclerosis. J. Neurosci. 29, 10272–10280. 10.1523/JNEUROSCI.1975-09.2009 19692601 PMC2747667

[B9] Das Sarma J. Scheen E. Seo S.-H. Koval M. Weiss S. R. (2002). Enhanced green fluorescent protein expression may be used to monitor murine coronavirus spread *in vitro* and in the mouse central nervous system. J. Neurovirol. 8, 381–391. 10.1080/13550280260422686 12402164 PMC7095158

[B10] Dubois-Dalcq M. E. Doller E. W. Haspel M. V. Holmes K. V. (1982). Cell tropism and expression of mouse hepatitis viruses (MHV) in mouse spinal cord cultures. Virology 119, 317–331. 10.1016/0042-6822(82)90092-7 6281976 PMC7111272

[B11] Gallagher T. M. Buchmeier M. J. (2001). Coronavirus spike proteins in viral entry and pathogenesis. Virology 279, 371–374. 10.1006/viro.2000.0757 11162792 PMC7133764

[B12] Grinspan J. B. Franceschini B. (1995). Platelet-derived growth factor is a survival factor for PSA-NCAM+ oligodendrocyte pre-progenitor cells. J. Neurosci. Res. 41, 540–551. 10.1002/jnr.490410414 7473886

[B13] Hemmer B. Archelos J. J. Hartung H.-P. (2002). New concepts in the immunopathogenesis of multiple sclerosis. Nat. Rev. Neurosci. 3, 291–301. 10.1038/nrn784 11967559

[B14] Hernán M. A. Zhang S. M. Lipworth L. Olek M. J. Ascherio A. (2001). Multiple sclerosis and age at infection with common viruses. Epidemiology 12, 301–306. 10.1097/00001648-200105000-00009 11337603

[B15] Kornek B. Storch M. K. Weissert R. Wallstroem E. Stefferl A. Olsson T.. (2000). Multiple sclerosis and chronic autoimmune encephalomyelitis: a comparative quantitative study of axonal injury in active, inactive, and remyelinated lesions. Am. J. Pathol. 157, 267–276. 10.1016/S0002-9440(10)64537-3 10880396 PMC1850217

[B16] Lassmann H. Brück W. Lucchinetti C. F. (2007). The immunopathology of multiple sclerosis: an overview.[see comment]. Brain Pathol. 17, 210–218. 10.1111/j.1750-3639.2007.00064.x 17388952 PMC8095582

[B17] Li J. Liu Y. Zhang X. (2010). Murine coronavirus induces type I interferon in oligodendrocytes through recognition by RIG-I and MDA5. J. Virol. 84, 6472–6482. 10.1128/JVI.00016-10 20427526 PMC2903279

[B18] Liu Y. Herbst W. Cao J. Zhang X. (2011). Deficient incorporation of spike protein into virions contributes to the lack of infectivity following establishment of a persistent, non-productive infection in oligodendroglial cell culture by murine coronavirus. Virology 409, 121–131. 10.1016/j.virol.2010.10.006 21035161 PMC3032362

[B19] Liu Y. Pu Y. Zhang X. (2006). Role of the mitochondrial signaling pathway in murine coronavirus-induced oligodendrocyte apoptosis. J. Virol. 80, 395–403. 10.1128/JVI.80.1.395-403.2006 16352564 PMC1317518

[B20] Major E. O. Amemiya K. Tornatore C. S. Houff S. A. Berger J. R. (1992). Pathogenesis and molecular biology of progressive multifocal leukoencephalopathy, the JC virus-induced demyelinating disease of the human brain. Clin. Microbiol. Rev. 5, 49–73. 1310438 10.1128/cmr.5.1.49 PMC358223

[B21] Marek R. Caruso M. Rostami A. Grinspan J. B. Das Sarma J. (2008). Magnetic cell sorting: a fast and effective method of concurrent isolation of high purity viable astrocytes and microglia from neonatal mouse brain tissue. J. Neurosci. Methods 175, 108–118. 10.1016/j.jneumeth.2008.08.016 18786564

[B22] Rudick R. A. Trapp B. D. (2009). Gray-matter injury in multiple sclerosis. N. Engl. J. Med. 361, 1505–1506. 10.1056/NEJMcibr0905482 19812410

[B23] Sharma R. Narayana P. A. Wolinsky J. S. (2001). Grey matter abnormalities in multiple sclerosis: proton magnetic resonance spectroscopic imaging. Mult. Scler. 7, 221–226. 10.1177/135245850100700402 11548980

[B24] Vercellino M. Plano F. Votta B. Mutani R. Giordana M. T. Cavalla P. (2005). Grey matter pathology in multiple sclerosis. J. Neuropathol. Exp. Neurol. 64, 1101–1107. 10.1097/01.jnen.0000190067.20935.42 16319720

[B25] Weiner L. P. Herndon R. M. Narayan O. Johnson R. T. Shah K. Rubinstein L. J.. (1972). Isolation of virus related to SV40 from patients with progressive multifocal leukoencephalopathy. N. Engl. J. Med. 286, 385–390. 10.1056/NEJM197202242860801 4333082

